# Rarity facets of biodiversity: Integrating Zeta diversity and Dark diversity to understand the nature of commonness and rarity

**DOI:** 10.1002/ece3.8096

**Published:** 2021-09-14

**Authors:** Federico Riva, Stefano Mammola

**Affiliations:** ^1^ Geomatics and Landscape Ecology Laboratory Department of Biology Carleton University Ottawa ON Canada; ^2^ Insectarium Montreal Space for Life Montreal QC Canada; ^3^ Laboratory for Integrative Biodiversity Research (LIBRe) Finnish Museum of Natural History (LUOMUS) University of Helsinki Helsinki Finland; ^4^ Molecular Ecology Group (MEG) Water Research Institute (IRSA) National Research Council (CNR) Pallanza Italy

**Keywords:** Alpha diversity, Beta diversity, biodiversity facets, commonness, diversity index, diversity metric, Gamma diversity, rarity

## Abstract

Measuring commonness and rarity is pivotal to ecology and conservation. Zeta diversity, the average number of species shared by multiple sets of assemblages, and Dark diversity, the number of species that could occur in an assemblage but are missing, have been recently proposed to capture two aspects of the commonness‐rarity spectrum. Despite a shared focus on commonness and rarity, thus far, Zeta and Dark diversities have been assessed separately. Here, we review these two frameworks and suggest their integration into a unified paradigm of the “rarity facets of biodiversity.” This can be achieved by partitioning Alpha and Beta diversities into five components (the Zeta, Eta, Theta, Iota, and Kappa rarity facets) defined based on the commonness and rarity of species. Each facet is assessed in traditional and multiassemblage fashions to bridge conceptual differences between Dark diversity and Zeta diversity. We discuss applications of the rarity facets including comparing the taxonomic, functional, and phylogenetic diversity of rare and common species, or measuring species' prevalence in different facets as a metric of species rarity. The rarity facets integrate two emergent paradigms in biodiversity science to better understand the ecology of commonness and rarity, an important endeavor in a time of widespread changes in biodiversity across the Earth.

## Graphical abstract

Despite a shared focus on commonness and rarity, Zeta diversity (average number of species shared by multiple sets of assemblages) and Dark diversity (species that could occur in an assemblage but are missing) have been thus far separate paradigms. We suggest integrating them into a unified framework of the “rarity facets of biodiversity” that integrate two emergent paradigms in biodiversity science to better understand the ecology of commonness and rarity. This is an important endeavor in a time of widespread changes in biodiversity across the Earth.

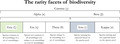

## 1 INTRODUCTION

Measuring biodiversity change is at the heart of ecology (MacArthur, 1965; Magurran, 2004; Whittaker, 1960), a property of considerable interest being how rare or common different biodiversity units are (Gaston, 2011; Preston, 1962). Rarity and commonness are important because they relate to several ecological processes, for example, community structure and trophic webs (Gaston, 2011; Violle et al., 2017), and because they are used to inform species management and conservation (IUCN, 2021). However, they are also multipronged concepts, often difficult to measure practically (Rabinowitz, 1981). Additionally, taxonomically rare species are not necessarily rare functionally or phylogenetically, and many metrics have been developed to evaluate different forms of rarity (Chao et al., 2014; Kondratyeva et al., 2019; Violle et al., 2017). Add to that how complex measuring biodiversity in the broad sense is (Daly et al., 2018), and it is not surprising that new approaches to measure rarity across the biodiversity facets (i.e., taxonomic, phylogenetic, and functional diversity; Chao et al., 2014; Pollock et al., 2020) are still being proposed and scrutinized after decades of work.

**FIGURE 1:**
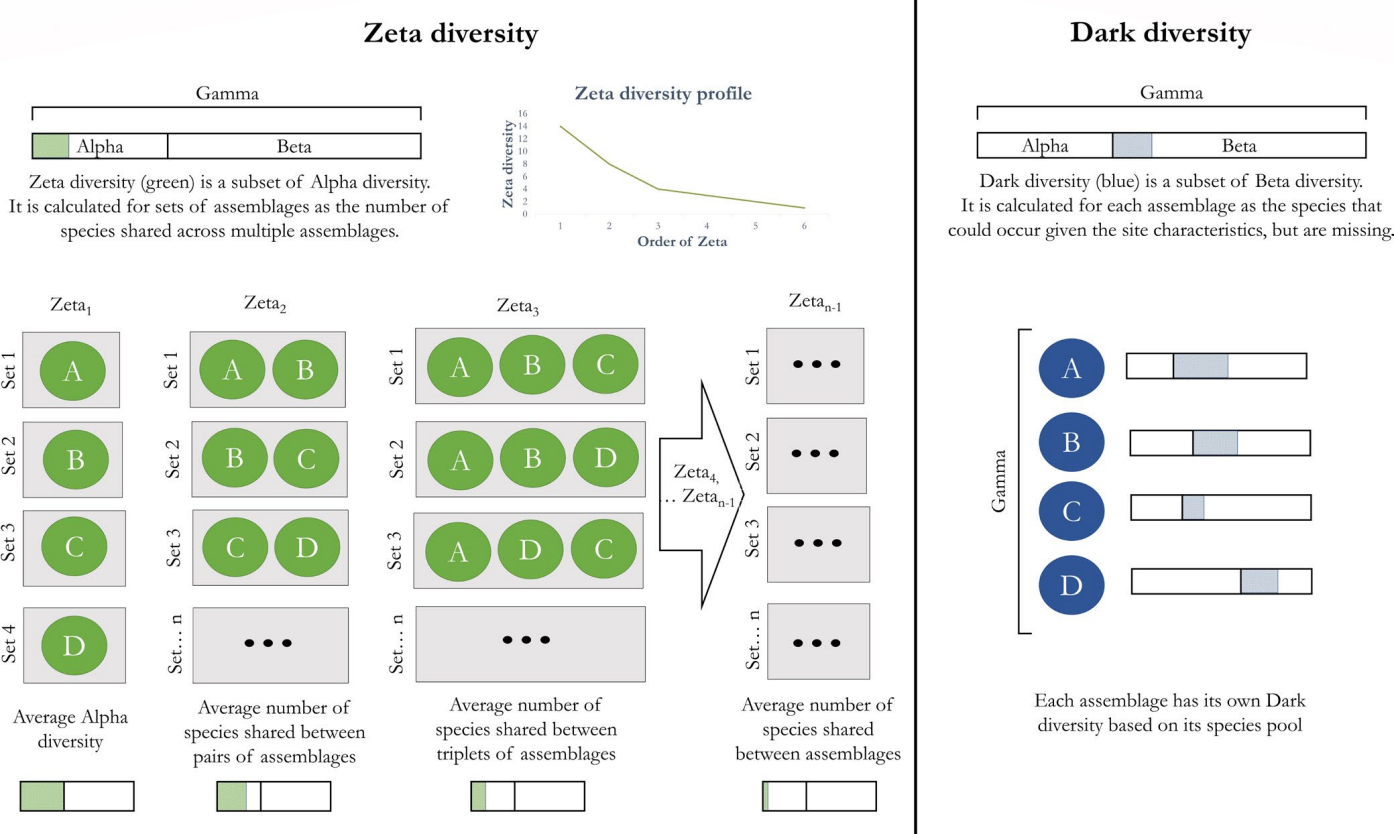
Summary of the Zeta and Dark diversities paradigms. Zeta diversity is outlined on the left, in green, whereas Dark diversity is outlined on the right, in blue. Gray rectangles represent sets of assemblages from which numbers of shared species are averaged to obtain Zeta diversity. Ellipses represent the scenarios missing from the rarity facets that are not shown in this illustrative figure

Recently, two metrics of taxonomic diversity were proposed to assess the rarity and commonness of species—Dark diversity (Pärtel et al., 2011) and Zeta (“ζ”) diversity (Hui & McGeoch, 2014). We contend that integrating these two paradigms within a more general heuristic, a single paradigm aimed at assessing the “rarity facets of biodiversity,” would aid in understanding the processes determining commonness and rarity of biodiversity units in nature. To achieve this synthesis, we propose to partition traditional Alpha and Beta diversities (Lande, 1996; Whittaker, 1960) in rarity components inspired by Zeta and Dark diversities, along a rarity‐commonness gradient. Zeta diversity and Dark diversity as originally defined are integrated within the framework as two of five rarity facets, with each facet being measured as multiassemblage, recursive series of indices, and as traditional diversity indices (following the original approaches proposed for Zeta diversity and Dark diversity, respectively; Figure 1). We discuss how assessing the rarity facets can inform about which processes structure species assemblages or define the rarity of a species, and conclude by providing a series of outstanding questions that can be addressed using the rarity facets. We provide ancillary information in three boxes to contextualize the rarity facets paradigm. In Box 1, we summarize how rarity has been conceptualized in ecology (for convenience, we use the term “rarity” from here on to refer to the rarity‐commonness spectrum). In Box 2, we summarize traditional approaches used to measure biodiversity, and how these relate to the rarity facets. Finally, in Box 3, we contextualize the rarity facets and discuss potential links with the biodiversity facets.

BOX 1 **On rarity**

Traditionally, there have been at least two perspectives when assessing rarity in ecology. Most agree that rarity is a property of biodiversity units, for example, one species can be rare in a set of sites if it occurs less commonly than the other species, or a trait can be rare when it is unique across a group of species. This definition, however, varies depending on the sites/assemblages under consideration and on how rarity is defined (Gaston, 2011; Magurran, 2004). The work of Rabinowitz (1981) is perhaps the most famous attempt to formalize the definition of what a “rare” species is, namely a species with small populations, small geographic ranges, and/or high habitat specificity. Rabinowitz's framework inspired much research on rarity across the three biodiversity facets (Kondratyeva et al., 2019).

Several ecologists also studied rarity as a property of communities, for example by measuring how many uncommon species are found in one or multiple assemblages (Pielou, 1966; Preston, 1962). This second perspective is generally represented with indices that describe or incorporate the preponderance of rare taxa in an assemblage. For instance, the relative abundance of diversity units can be accounted for when measuring biodiversity (Chao et al., 2014) such that two sites with an equal number of species can differ in their diversity depending on the species–abundance distribution (Roswell et al., 2021). This second approach ignores the taxonomic identity of species.

Here, we refer to rarity as a property of biodiversity units, following the first school of thought. However, we note that the species–abundance distribution within each rarity facet can be in principle assessed in the rarity facets (e.g., using Hill numbers to describe the assemblages typical of each rarity facet; see “outstanding questions” in Table 1 and Box 3). Therefore, the rarity facets have the potential to bridge the two major perspectives on rarity in ecology.

**TABLE 1:** Outstanding research questions that can be addressed by assessing the rarity facets of biodiversity

Question	Approach	Application
How rare is a species, and what type of rarity?	By assessing the prevalence of a species in each of the five rarity facets. Rare species will be more likely to belong to Theta and Iota. When considering abundance data and the spatial extent of the data analyzed, the rarity facets can be used to classify species in the rarity forms proposed by Rabinowitz (1981)	Classifying species according to their rarity (e.g., cluster analysis of species similarity based on their relative occurrence in different rarity facets)
How do the commonness and rarity of species vary through space and time?	By comparing the rarity facets of different assemblages or of the same assemblages before and after a disturbance event or along a temporal series	Understanding the implications of habitat change (e.g., due to anthropogenic and natural disturbance regimes) for conservation
What are the taxonomic, phylogenetic, and functional characteristics of common and rare species? To which degree are they correlated?	Comparing the taxonomic, functional, and phylogenetic diversity of species belonging to different rarity facets	Facilitate the identification of taxa of conservation concern based on general patterns (e.g., relationship between body size and rarity)
What is the relationship between adaptive strategy and rarity?	By comparing taxonomic groups differing for a character of interest (e.g., dispersal) across the same set of assemblages	Assess which processes relate to differences between taxa inhabiting the same system
What are the community assembly mechanisms that most influence the commonness‐rarity gradient?	By comparing the distribution of rarity components among ecological systems with different selective regimes	Understanding the origin of rarity in different taxonomic groups or habitats
How much variation in commonness and rarity is there across a set of assemblages?	By assessing the variation around the mean index value in the multiassemblage indices (e.g., the standard deviation of Zeta_1,2,3…_ *_n_*_−1_)	Inferring the degree of specialization of different taxonomic groups inhabiting the same habitat
What is the relation between rarity and the species–abundance distribution?	By assessing the species–abundance distribution in different rarity facets, for example, by measuring evenness or using Hill numbers on abundance data	Understanding the origin of rarity
Which assemblages should we protect if the goal is maximizing the protection of rare species?	By selecting sites with the highest Theta_0_ and lowest Iota_0_	Supporting conservation planning and landscape prioritization
When do different rarity facets correlate positively and negatively?	By comparing trends in the rarity facets across many different ecological systems and taxa	Discovering patterns of correlation between commonness and rarity to inform conservation

BOX 2 **Fundamentals of measuring biodiversity and rarity**

Measuring biodiversity patterns is one of the most important endeavors in ecology and evolution (Magurran, 2004). At the highest conceptual level, diversity metrics have been categorized based on three aspects of biodiversity—the taxonomic, functional, and phylogenetic diversity facets (Chao et al., 2014; Kondratyeva et al., 2019; Mammola et al., 2021; Pollock et al., 2020). Within these three biodiversity facets, tens of approaches have been proposed to measure differences between groups of species (Ellison, 2010; Hurlbert, 1971); some can be used to assess biodiversity across all facets (Chao et al., 2014; see Box 3).

**FIGURE 2:**
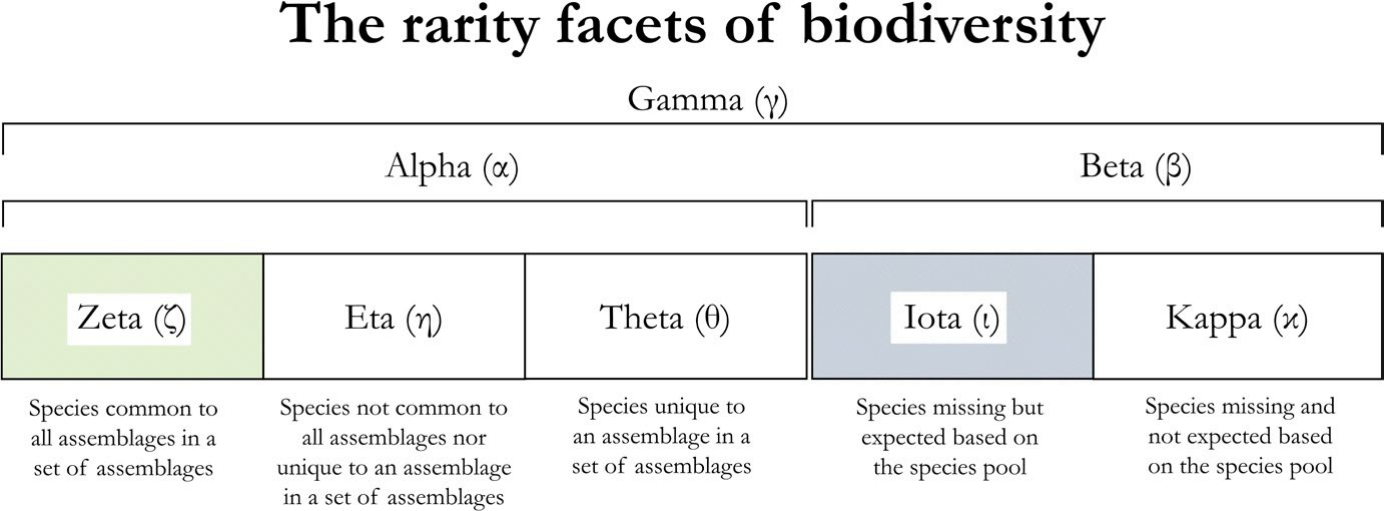
The five rarity facets of biodiversity are proposed as subsets of traditional Alpha and Beta diversities (see Box 2), based on the commonness and rarity of species. Colors (green and blue) connect the Zeta and Iota diversity facets with the Zeta and Dark diversity paradigms outlined in Figure 1

**FIGURE 3:**
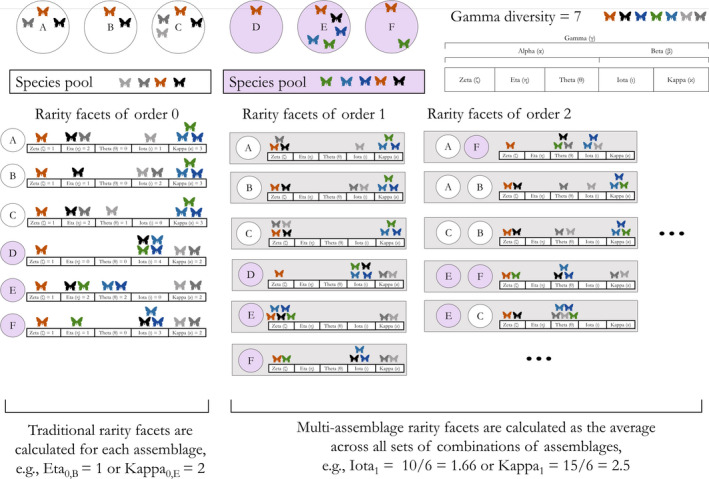
An example of how the rarity facets are calculated based on six assemblages of butterflies from two different species pools. On the top of the figure, seven species (different colors) are shown within the six assemblages (A–F). Species' clockwise order facilitates distinguishing species shared between assemblages. On the lower side of the figure, we show how each rarity facet would be calculated for the traditional metrics (Rarity facets of order 0) and for an example of the first order of the multi assemblage metrics (Rarity facets of order 1). Ellipses represent the scenarios missing from the rarity facets of order 2, and additional rarity facets (here up to the 5th order) that are not shown in this illustrative figure

Another seminal paradigm defined the decomposition of biodiversity indices in components representing different properties of a set of sites. The rarity facets (Figure 2) belong to this family of decomposition metrics and can be applied to the three biodiversity facets described above. The rarity facets follow perhaps the most famous decomposition paradigm, which was defined by Robert Whittaker in the 1960s (Ellison, 2010; Whittaker, 1960). The overarching idea is that, in a set of species assemblages, we can evaluate how biodiversity scales from smaller (Alpha diversity) to larger scales (Gamma diversity) by assessing the mathematical relationship between these two measures (Beta diversity). Whittaker's idea is appealing for generality and simplicity, but it is open to interpretation. For instance, both additive (Alpha + Beta = Gamma) and multiplicative (Alpha * Beta = Gamma) Beta diversity propositions exist in the literature (Ellison, 2010; Jost, 2007; Lande, 1996). Additionally, Whittaker initially defined Alpha as a property of each of many assemblages (i.e., the number of species at each sampling location; Whittaker, 1960), and later as a property of sets of assemblages (i.e., the average number of species across all locations; Whittaker, 1972). Here, we adopt Whittaker's first definition (1960), assuming that Alpha and Beta diversities are site‐specific, because we are interested in maintaining the identities of the species belonging to each rarity facet in each site (Box 3). Note that this is also how Pärtel et al. (2011) intended Alpha and Beta diversity in their seminal paper on Dark diversity. Therefore, the rarity facets represent subsets of Alpha (Zeta, Eta, and Theta), and of Beta (Iota and Kappa) (Figures 2,3). When considering incidence data, the rarity facets also follow an additive partitioning rule, such that the rarity facets within Alpha and Beta add up to Gamma in an additive fashion (Figure 2).

BOX 3 **Measuring “the biodiversity facets of the rarity facets”**

We suggest that one intriguing application of the rarity facets would be assessing “the biodiversity facets of the rarity facets.” This approach could aid in answering some of the outstanding questions proposed in Table 1. For example, one could use Hill numbers to calculate the functional, phylogenetic, and taxonomic diversities of Zeta, Eta, Theta, Iota, and Kappa (Figures 2, 3) and compare the properties of species found in different rarity facets to infer how functional and phylogenetic characteristics relate to commonness and rarity of species. We suggest specifically Hill numbers because this paradigm has been proposed as the unifying framework for measuring biodiversity (Chao et al., 2014; Ellison, 2010; Roswell et al., 2021), but other approaches are indeed possible.

We named the rarity facets following the Greek letters Zeta, Eta, Theta, Iota, and Kappa because (a) they recall Alpha and Beta diversity, and (b) they are not associated with widespread paradigms in biodiversity science. We recognize that these letters might have been used in the past in similar contexts. For example, Scalercio et al. (2007) used “eta diversity” to refer to the concept of “ecotope species richness.” We hope that using the five rarity facets together will reduce the risk of confusion with other attempts of defining different concepts using only one of these five letters.

## 2 WHAT ARE ZETA DIVERSITY AND DARK DIVERSITY?

Zeta diversity and Dark diversity are metrics developed to assess two aspects of the commonness and rarity of species across a group of assemblages using incidence (presence/absence) data (Figure 1).

Zeta diversity (ζ_i_) was defined as “the number of species shared by multiple assemblages” (Hui & McGeoch, 2014). Specifically, it is a family of indices defined by the number of species shared across many assemblages, whereby ζ_1_ is the average number of species across a set of assemblages, ζ_2_ is the average number of species shared by any two assemblages of the set, ζ_3_ is the average number of species shared by any three assemblages of the set, and so on (ζ_4_ … ζ*_n_*_−1_, where n is the total number of assemblages). Zeta diversity is generally shown as diversity profiles that decrease monotonically with increasing order of Zeta (i.e., number of assemblages included in the calculation; Figure 1).

Dark diversity was defined as the “species that are absent from an ecosystem, but which belong to its species pool” (Pärtel et al., 2011). It is a refinement of the species pool hypothesis (Cornell & Harrison, 2014), representing the subset of Beta diversity (sensu Whittaker, 1960) that could inhabit a site based on its ecological characteristics, but does not.

Dark and Zeta diversities are measured in different ways (Figure 1), and we develop the framework of the rarity facets of biodiversity to capitalize on both approaches. Specifically, Zeta diversity introduced a novel multiassemblage recursive approach to measure diversity across sets of assemblages. This idea emerged because traditional metrics are inadequate to describe properties of sets of assemblages (Hui & McGeoch, 2014), and allows assessing how the number of species shared among assemblages varies when considering different numbers of assemblages. In other words, Zeta diversity is not a single metric but rather a family of metrics that vary with the number of assemblages included in the calculation, such that the higher the order of Zeta, the more common a species has to be to be shared between assemblages. Conversely, Dark diversity belongs to a family of traditional diversity indices designed to measure the diversity of an assemblage and/or to compare pairs of assemblages (Hui & McGeoch, 2014). The most commonly used diversity indices, like species richness or Shannon entropy (Jost, 2006; Roswell et al., 2021), belong to this type of diversity metrics, with many applications including partitioning of Gamma diversity in Alpha and Beta components (Lande, 1996; Whittaker, 1960; Box 1).

## 3 WHY SHOULD WE INTEGRATE ZETA DIVERSITY AND DARK DIVERSITY?

Zeta diversity and Dark diversity have been so far assessed separately. For instance, searching for the string “zeta diversit*” AND “dark diversit*” we found no matches in the Web of Science database (search conducted on 2 August 2021). However, we contend that they can be seen as complementary facets of one paradigm, because both approaches focus on different aspects of the commonness and rarity of species. Indeed, the more common a species is, the more likely it will be to belong to high orders of Zeta diversity (Latombe et al., 2017), whereas the rarer one species is, the more likely it will be to belong to Dark diversity (Lewis et al., 2017). Furthermore, both paradigms focus on subsets of one or more assemblages—species that are common to more assemblages (Zeta diversity), and species that are absent but could occur in an assemblage (Dark diversity). Zeta and Dark diversities also share similar applications, for example, understanding trade‐offs between local and regional processes in structuring species assemblages (Pärtel et al., 2011) or the mechanisms underlying species turnover (Hui & McGeoch, 2014; McGeoch et al., 2019), and of course the study of species rarity for ecological and conservation applications (Latombe et al., 2017; Lewis et al., 2017; Pärtel et al., 2011). Ultimately, despite differing in how they are calculated and in what they measure, there is broad overlap in the concepts underlying Dark and Zeta diversities. A synthesis of these concepts would facilitate progress in the study of commonness and rarity in natural systems.

## 4 THE RARITY FACETS OF BIODIVERSITY

We propose to decompose traditional Alpha (α) and Beta (β) diversities in five rarity facets, three facets within Alpha [Zeta (ζ), Eta (η), and Theta (θ)] and two within Beta [Iota (ι) and Kappa(κ)] (Figure 2). Each facet could be measured as a traditional metric (indicated with a subscript zero, e.g., Theta_0_) and/or as multiassemblage metrics (indicated with progressive subscripts, e.g., Theta_1_, Theta_2_, Theta_3_ … Theta*_n_*_−1_, where n is the total number of assemblages considered in the analysis). The five rarity facets include traditional Dark and Zeta diversities and are defined as follows:

Components of Alpha:

‐ Zeta_i_: species that are common to all assemblages; Zeta_i_ corresponds to Zeta diversity sensu Hui and McGeoch (2014) when calculated as a multiassemblage metric (*i* > 0).
‐ Eta_i_: species that are not unique to an assemblage nor common to all assemblages.
‐ Theta_i_: species that are unique to an assemblage.

Components of Beta:

‐ Iota_i_: species expected based on the species pool, but that were not observed; Iota corresponds to Dark diversity sensu Pärtel et al. (2011) when calculated as a traditional metric (*i* = 0).
‐ Kappa_i_: species present in a set of assemblages, but that are not expected in a specific assemblage.

We show in Figure 3 a toy example of the paradigm application. When estimated as traditional metrics, as in the Dark diversity framework (Pärtel et al., 2011), each rarity facet is calculated for each assemblage in relation to the entire set of assemblages and to its species pool (e.g., Zeta_0_ is equal in all assemblages, whereas Theta_0_ is unique to each assemblage). When calculated as multiassemblage metrics, as in the original Zeta diversity framework (Hui & McGeoch, 2014), the rarity facets correspond to the average number of species that occur in the five rarity facets across the possible combinations of *n* assemblages from the full set of assemblages (Figure 3, gray rectangles; e.g., Kappa_3_ is the average number of species present in the study region but that are missing from sets of three assemblages because they do not belong to the species pool of those assemblages). Following Hui and McGeoch (2014), Zeta_1_ is equal to the average Alpha diversity, because all species are “common” to the assemblage when each assemblage is compared with itself. One could argue that all species are also “unique” to the assemblage in this particular case; we chose to define Theta_1_ equal to zero because we believe that it is more intuitive, and to maintain complementarity of the rarity facets. Note that Eta_1_ and Eta_2_ are also always equal to zero by definition, because in an assemblage or between two assemblages there cannot be species that are not shared or unique to one of the two assemblages.

## 5 DISCUSSION

We proposed the rarity facets of biodiversity as a framework aimed at better understanding what determines the commonness and rarity of biodiversity units. We did so by synthesizing in a cohesive paradigm Dark diversity (Pärtel et al., 2011) and Zeta diversity (Hui & McGeoch, 2014). In our estimation, there is no reason to limit the approaches proposed for Zeta diversity and Dark diversity only to the subsets of species shared in sets of assemblages, or missing from an assemblage despite belonging to its species pool (Figure 1). Studying the rarity facets together, rather than independently and partially as is currently done in studies that use Zeta diversity and Dark diversity, will aid ecologists in seeking a deeper understanding of the commonness‐rarity spectrum because it allows to take full advantage on the strengths of both approaches. For instance, traditional (order 0) metrics can be used to compare individual sites or to contrast local versus. regional rarity patterns, and multisite (order 1… *n* − 1) metrics used to seek a broader understanding of the processes that shape commonness and rarity within a region or among different regions.

We outline the framework exclusively on a theoretical ground, but we anticipate applications as diverse as the field of ecology itself (Table 1). For instance, one could ask whether and how commonness and rarity of species vary in space, time, and across scales (McGill, 2010), both at local and at regional scales (Figure 3). Additionally, while we described the framework using incidence data and in the taxonomic diversity facet for simplicity and for consistency with the original Zeta and Dark diversities, the approach can be easily expanded to the other diversity facets and to abundance data (see Box 3). Indeed, one could estimate phylogenetic or functional diversity for the subassemblages of species within each rarity facet, for example by using Hill numbers (Chao et al., 2014; Roswell et al., 2021; Box 3), or could evaluate whether rare or common species tend to be more or less evenly distributed in abundance (Pielou, 1966; Box 1). Furthermore, analyzing abundance data one could classify species according to Rabinowitz's seven forms of rarity (i.e., based on their rarity and abundance; Table 1). Ultimately, the rarity facets paradigm lends itself to answer a variety of basic and applied questions across the three biodiversity facets. It goes beyond the use of Zeta and Dark diversity as independent paradigms because it allows evaluating rare and common species together, providing comprehensive information on the characteristics of each of many sites, as well as on the characteristics of a set of sites (Figure 3). It also has the potential to bridge two schools of thought in the study of rarity, because it allows assessing the rarity of species and the species–abundance distribution simultaneously (Box 1).

Developing a tool to measure the rarity facets of biodiversity is the natural step following this conceptual paper. However, it involves complications that we are currently addressing—particularly for multiassemblage metrics. Answering many outstanding questions requires maintaining the identity of the species belonging to each rarity facet in each set of assemblages, such that estimating the rarity facets mathematically as proposed for Zeta diversity (Hui & McGeoch, 2014) is not suitable. Simulating sets of assemblages as originally proposed for Zeta diversity (Hui & McGeoch, 2014), rather than calculating all possible combinations of n sites for each rarity facet (which is computationally demanding for large datasets), might aid in bypassing the curse of dimensionality (Bellman, 1957). Furthermore, defining the species pool of assemblages (Lewis et al., 2017) or what exactly is habitat for a set of species (Riva & Nielsen, 2020) can be difficult. Therefore, actualizing the rarity facets paradigm will require robust technical and theoretical backgrounds.

Ultimately, here we highlighted that Zeta and Dark diversity can be seen as two aspects of a single paradigm, suggesting a novel perspective to understand the ecology of outliers (Violle et al., 2017) and of common species (Gaston, 2011). Commonness and rarity are properties of historical interest (Preston, 1962), but monitoring their change is also a timely conservation endeavor amidst the current biodiversity crisis (Ceballos et al., 2017). Indeed, taxa are declining not only in species that are historically considered of conservation concern, but also in more widespread species (Forister et al., 2021; Rosenberg et al., 2019). Monitoring how commonness and rarity change in space and time will be paramount to inform effective management practices, and assessing the rarity facets could reveal how commonness and rarity of species are changing in the Anthropocene, across the three diversity facets (Chao et al., 2014), and accounting for changes in species' abundances (Preston, 1962). All these aspects are crucial to effective biodiversity conservation (Pollock et al., 2020). We hope that this manuscript will stimulate advancements in the study of commonness and rarity, while paving the way to the technical development of the new paradigm of the rarity facets of biodiversity.

## CONFLICT OF INTEREST

None declared.

## AUTHOR CONTRIBUTIONS

**Federico Riva:** Conceptualization (equal); Methodology (lead); Visualization (lead); Writing‐original draft (lead). **Stefano Mammola:** Conceptualization (equal); Methodology (supporting); Writing‐original draft (supporting).

## Data Availability

No data were analyzed in this conceptual piece.
